# Autonomic modulation by SGLT2i or DPP4i in patients with diabetes favors cardiovascular outcomes as revealed by skin sympathetic nerve activity

**DOI:** 10.3389/fphar.2024.1424544

**Published:** 2024-07-30

**Authors:** Jien-Jiun Chen, Chen Lin, Men-Tzung Lo, Lian-Yu Lin, Hsiang-Chih Chang, Geng-Chi Liu

**Affiliations:** ^1^ Department of Internal Medicine, Division of Cardiology, Yunlin Branch of National Taiwan University Hospital, Yunlin, Taiwan; ^2^ Department of Biomedical Sciences and Engineering, National Central University, Taoyuan, Taiwan; ^3^ Department of Internal Medicine, Division of Cardiology, College of Medicine, National Taiwan University and Hospital, Taipei, Taiwan

**Keywords:** NeuECG, skin sympathetic nerve activity, autonomic modulation, entropy analysis, sodium-glucose cotransporter 2 inhibitor

## Abstract

**Background:**

Sodium-glucose cotransporter 2 inhibitors (SGLT2i) and dipeptidyl peptidase-4 inhibitors (DPP4i) are important second-line treatments for patients with type 2 diabetes mellitus (T2DM). Patients taking SGLT2i have favorable cardiovascular outcomes via various mechanisms, including autonomic nervous system (ANS) modulation. This study aimed to use neuro-electrocardiography (neuECG) to test the effects of SGLT2i or DPP4i on the ANS.

**Methods:**

Patients with T2DM, who did not reach target hemoglobin (Hb)A1C levels despite metformin treatment, were enrolled. SGLT2i or DPP4i were prescribed randomly unless a compelling indication was present. NeuECG and heart rate were recorded for 10 min before and after a 3-month treatment. The patients were treated according to standard practice and the obtained data for skin sympathetic nerve activity (SKNA) and ANS entropy were analyzed offline.

**Results:**

We enrolled 96 patients, of which 49 received SGLT2i and 47 received DPP4i. The baseline parameters were similar between the groups. No adverse event was seen during the study period. In the burst analysis of SKNA at baseline, all parameters were similar. After the 3-month treatment, the firing frequency was higher in SGLT2i group (0.104 ± 0.045 vs 0.083 ± 0.033 burst/min, p < 0.05), with increased long firing duration (7.34 ± 3.66 vs 5.906 ± 2.921, p < 0.05) in 3-s aSKNA scale; the other parameters did not show any significant change. By symbolic entropy, the most complex patterns (Rank 3) were found to be significantly higher in SGLT2i-treated patients than in DDP4i-treated group (0.084 ± 0.028 vs 0.07 ± 0.024, p = 0.01) and the direction of change in Rank 3, after SGLT2i treatment, was opposite to that observed in the DDP4i group (0.012 ± 0.036 vs. −0.005 ± 0.037, p = 0.024). Our findings demonstrated the favorable autonomic modulation by SGLTi and the detrimental effects of DPP4i on ANS.

**Conclusion:**

We demonstrated the autonomic modulation by SGLTi and DPP4i using SKNA in patients with DM, which might provide insights into the favorable outcomes of SGLT2i. Furthermore, we refined the analytical methods of neuECG, which uses SKNA to evaluate autonomic function.

## Introduction

Patients with type 2 diabetes mellitus (T2DM) have to take oral hypoglycemic agents in addition to lifestyle modifications (Cheng and Fantus, 2005). According to the current guidelines, the first-line therapy for T2DM uses metformin, followed by either sodium-glucose cotransporter 2 inhibitor (SGLT2i), dipeptidyl peptidase-4 inhibitor (DPP4i), α-glucosidase inhibitor, or sulfonylureas (Qaseem et al., 2017; Flory and Lipska, 2019; Grant and Cosentino, 2019; Dong et al., 2023). Of the second-line drugs, SGLT2i and DPP4i have no side effect of hypoglycemia (Molina-Vega et al., 2018), and have a positive effect (SGLT2i) on cardiovascular (CV) outcomes, including CV mortality, heart failure hospitalization, and myocardial infarction, regardless of the glycemic status (Zelniker et al., 2019; Chan and Chan, 2023; He et al., 2023; Talha et al., 2023). Although the CV effect of DPP4i is controversial, most studies have shown neutral effects (Scheen, 2018; Rosenstock et al., 2019; Subrahmanyan et al., 2021). The mechanisms underlying SGLT2i have favorable CV outcomes, including natriuresis, diuresis, glycemic control, improved cardiac metabolism, prevention of adverse cardiac remodeling and ischemia-reperfusion injury, and inhibition of sympathetic nervous system (SNS) activity (Cowie and Fisher, 2020; Lopaschuk and Verma, 2020). A previous study had compared heart rate variability (HRV) outcomes in patients with acute myocardial infarction (AMI) taking SGLT2i or placebo and showed that early SGLT2i administration in patients with AMI and T2DM might be effective in improving cardiac nerve activity without any adverse event (Shimizu et al., 2020). The study used traditional HRV to evaluate the SNS activity. Many theories have been postulated regarding this phenomenon, one such suggested that a reduction in renal stress results in the inhibition of renal afferent sympathetic activation (Sano, 2018; Herat et al., 2020; Sano, 2020).

We consider signals with frequencies beyond 150 Hz in the electrocardiography (ECG) as noise (Uradu et al., 2017). However, studies have shown that such signals can convey information about the skin sympathetic nerve activity (SKNA) (Jiang et al., 2015), and SKNA elevation or bursts have been found to trigger ventricular arrhythmia and initiate and terminate atrial arrhythmia (Kusayama et al., 2019). SKNA can be directly and noninvasively measured using conventional ECG electrodes with a high-band-pass amplifier (Kusayama et al., 2019). Validation was performed by recording stellate ganglion and thoracic invasion, which showed consistent data (Jiang et al., 2015). Standard methods for recording and analyzing these complex signals, including iSKNA and aSKNA, have already been published and formally named as neuECG (Kusayama et al., 2020). Although SKNA has been demonstrated to accurately estimate sympathetic nerve activity and be potent for clinical applications, there has been no report concerning the direct measurement of sympathetic activity in patients with T2DM taking second-line medication for which decreased cardiovascular morbidity and mortality and improved renal outcomes due to sympathetic inhibition have been emphasized (Karakas et al., 2013; Cowie and Fisher, 2020; Lopaschuk and Verma, 2020).

In this study, we considered that SKNA detected by neuECG can provide information on the sympathetic control of the cardiovascular and renal systems, resulting in favorable outcomes. We analyzed SKNA data using published methods and developed entropy analyses. To balance glycemic control as a possible confounding factor, we recruited patients taking DPP4i for comparison. We hypothesized that SGLT2i has favorable sympathetic modulation effects, which could be proven by SKNA using neuECG.

## Materials and methods

From December 2020 to August 2022, we recruited patients with T2DM who had hemoglobin A1C (HbA1C) levels higher than the therapeutic goal (6.5%) despite treatment with metformin or those who could not tolerate metformin. Patient was screened for eligibility and were excluded if he/she had heart failure symptoms or severe chornic kidney disease. The patients were prescribed SGLT2i (empagliflozin 25 mg once daily) or DPP4i (linagliptin 5 mg once daily) as second-line treatment for their DM per treating physician’s judgement (in a random manner unless compelling indication or contraindication existed).

### Data acquisition

**FIGURE 1:**
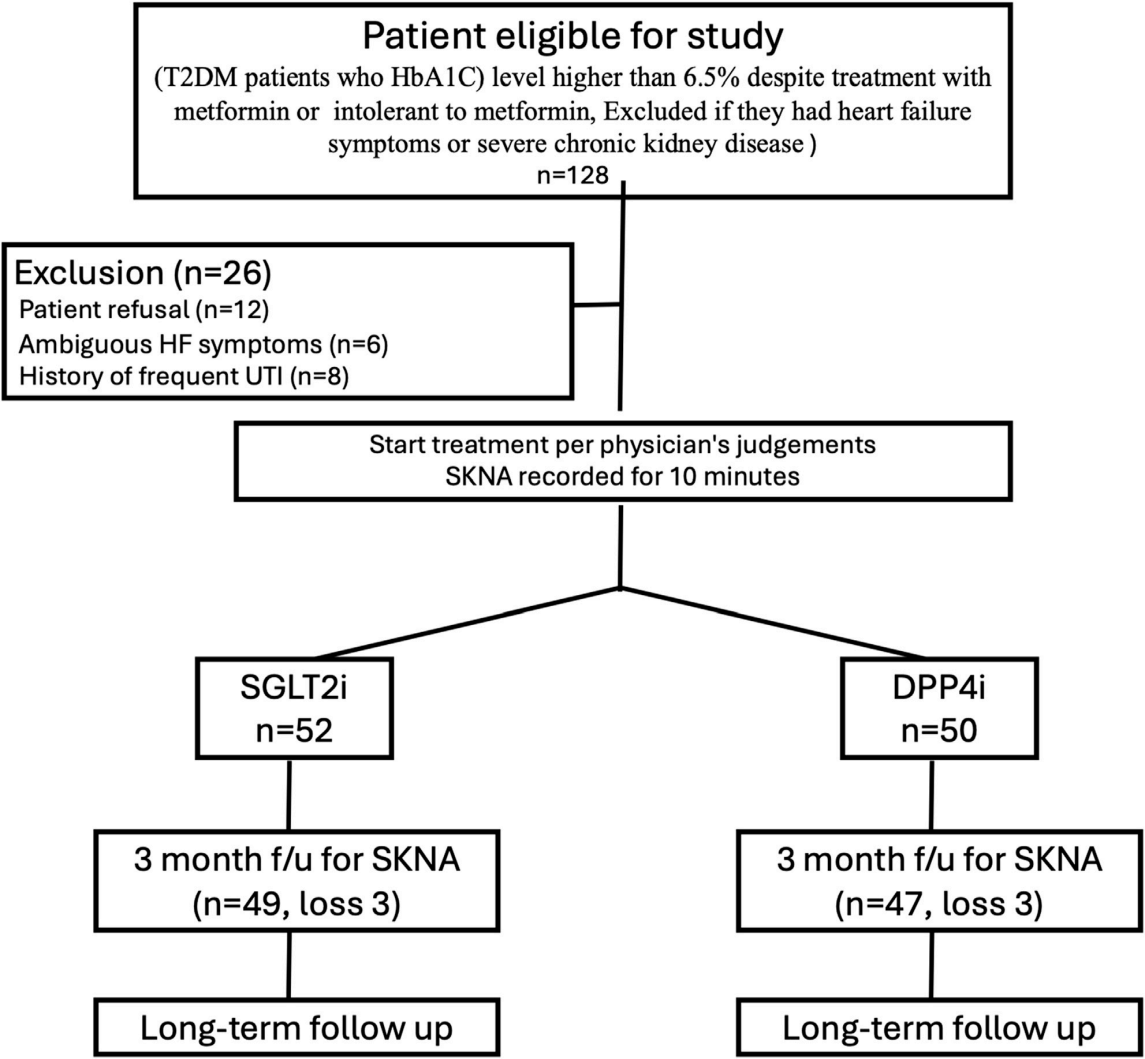
Study protocol. The figure showed the study protocol. After assessment of eligibility (Patients with T2DM, who had hemoglobin A1C (HbA1C) levels higher than the therapeutic goal (6.5%) despite treatment with metformin or those who could not tolerate metformin. Patient were excluded if he/she had heart failure symptoms or severe chornic kidney disease). We excluded some patients before prescription and performing SKNA recording. We excluded patients with symptoms that might be related to heart failure and patients with frequent urinary tract infection. Some patients were excluded because he/she did not want to take part in the study.

SKNA parameters were obtained using traditional bipolar electrodes, and Lead II signals were recorded for 10 min and digitized at 10,000 Hz by a fully programmable amplifier/data acquisition unit (MP36, BIOPAC, USA). Both ECG and SKNA data were filtered through two distinct filter types. Before treatment, we recorded the SKNA for 10 min, and after 3 months of treatment, we recorded the SKNA again. (Figure 1). All recordings were performed at an outpatient clinic in a small private room after the patient had rested fully. Drug compliance was confirmed by direct confrontation. Patient baseline characteristics, including underlying disease, glucose level, HbA1C level, renal function, and medication, were reviewed. Patients received usual care for their diseases. Offline analysis of the SKNA data was performed using custom-developed MATLAB programs. The study was conducted in compliance with the Declaration of Helsinki. This study was approved by the Research Ethics Committee of the National Taiwan University Hospital (202107096RINA).

### Data preprocessing

ECG recordings in adults were extracted using a band-pass filter ranging from 0.05 to 150 Hz, with amplitudes measured in millivolts. For the SKNA signal, a high-pass filter with a cutoff frequency of 500 Hz was employed to differentiate electrical signals from sources such as the electrocardiogram and myopotential signals. In addition, a 1000-Hz low-pass filter was used to attenuate the electrical noise caused by radio frequency interference (Kusayama et al., 2020; Chen et al., 2022). The filtered signals (SKNA) were then rectified. Summation of the instantaneous rectified SKNA over a 100-m time window was denoted as the integrated SKNA (iSKNA) (Kusayama et al., 2020; Chen et al., 2022). Averaging the iSKNA (aSKNA) signal over a less-than-2-s window significantly correlated with heart rate (i.e., heart rates significantly affected aSKNA amplitude due to similar frequency). Consequently, we considered only time windows of 3 s or longer as potential signals for further analysis, aiming to quantify time-dependent sympathetic nerve activity. Drawing inspiration from the frequency-domain analysis in heart rate variability tests, we hypothesized that shorter time windows are more likely to evaluate the interaction between sympathetic and parasympathetic activities. Time windows of 10 s or longer were deemed inapplicable for further analysis owing to the limited recording time. Additionally, the peak of each QRS wave in the ECG data was annotated using an automated algorithm and carefully corrected by experienced technicians.

### Burst analysis

The presence of aSKNA bursts may be related to the initiation and termination of cardiac arrhythmias (Sano, 2020). Burst activity can be differentiated from baseline activity, which consists of random single spikes with large amplitudes and durations. To differentiate between the baseline and burst, we used the k-means algorithm to cluster the unsupervised data (aSKNA) into two groups (Kusayama et al., 2020). The mean value plus three times the standard deviation of the group with a relatively low amplitude was used as the threshold for burst determination of each subject (Kusayama et al., 2020). A complete burst starts at the point where the amplitude of an aSKNA signal exceeds the threshold and ends at the point where the amplitude of the signal is below the threshold. The aSKNA parameters were calculated as follows:

- Burst amplitude (μV) is the average voltage during burst within the time frame being evaluated;
- Burst frequency (bursts/min) is the total number of “On,” amplitudes of aSKNA that exceed the threshold, in every minute;
-
$B\text{urst duration }\left(\%\right)=\frac{\text{total}\;\text{span}\;\text{of}\;\text{burst}\;\left(\min\right)}{\text{total}\;\text{span}\;\text{of}\;\text{the}\;\text{time}\;\text{frame}\;\text{being}\;\text{evaluated}\;\left(\min\right)}\times 100$ ;
  ♦ The study duration was divided into short and long periods. Long firing duration in 1-s window means >2 s and in 5-s window means > 10 s.
- Total burst area (μV · min) is the sum total of burst area (μV · min) within the time frame being evaluated.

### Symbolic entropy analysis

**FIGURE 2:**
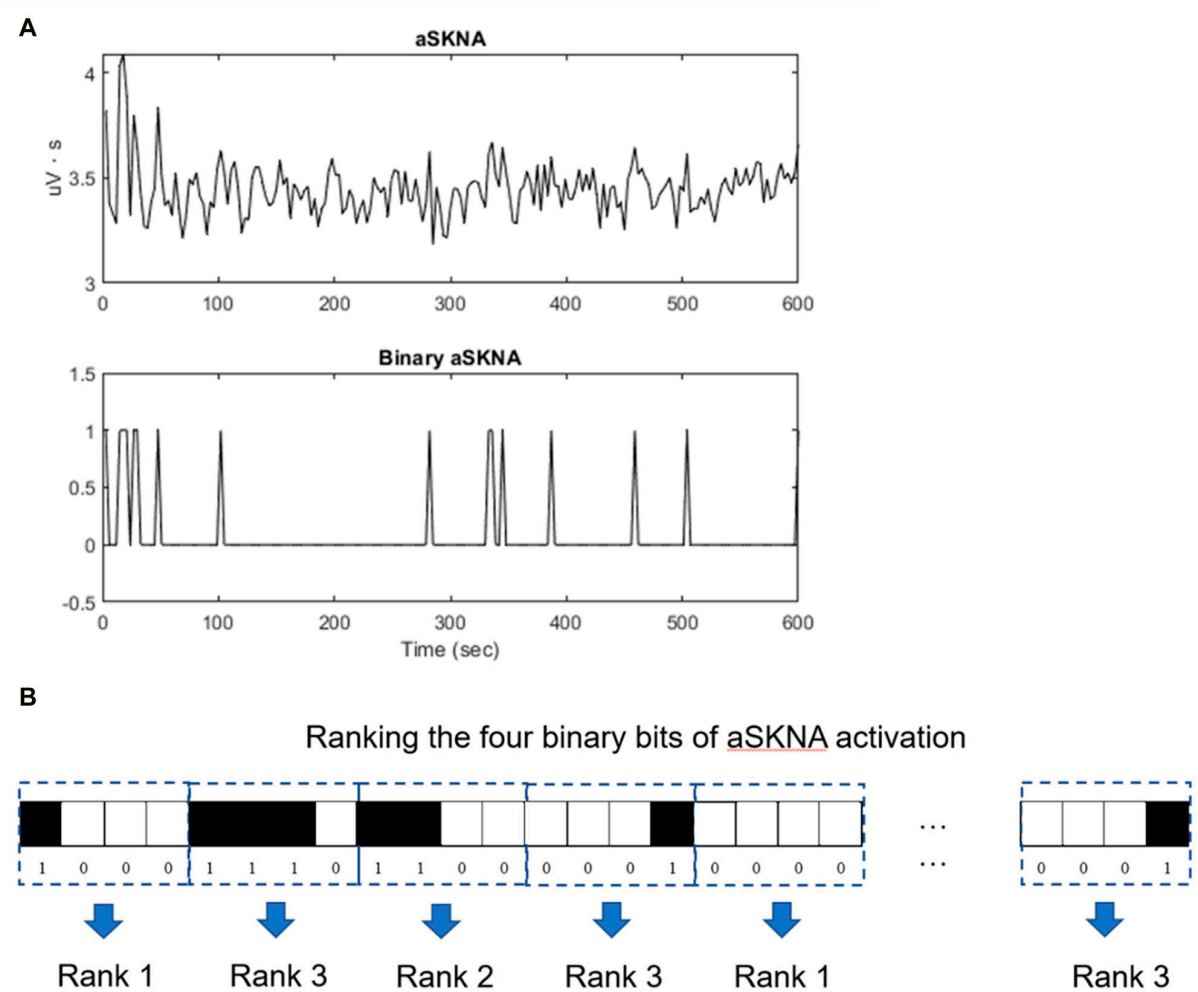
Entropy analysis of the aSKNA signals. **(A)** The aSKNA signal is transformed into a binary sequence by applying a specific threshold, “1” denoting activation, and “0” denoting non-activation. **(B)** Subsequently, the binary sequence is segmented into numerous non-overlapping blocks, each of uniform length, denoted by “m” (for instance, m = 4). These segments are classified into distinct categories according to their conditional probabilities, ensuring that segments within the same category possess identical conditional probabilities. Comprehensive categorization is performed for all conceivable 4-bit sequences, which are then arranged according to their conditional probabilities. For instance, a category ranked ’3′ represents sequences with the highest complexity, whereas a rank of ’1′ corresponds to sequences that are the most predictable.

To quantify the predictability or complexity of a highly fluctuating time series, we denoted the aSKNA signal as a binary sequence based on an individual threshold (1 for activation and 0 for non-activation). This sequence was then divided into multiple nonoverlapping segments of equal length m (in this study, m was set to 4). These m-bit sequences were categorized based on their temporal patterns using the concept of approximate/sample entropy (Lake et al., 2002; Lo et al., 2015). For instance, a 4-bit sequence was dissected into multiple single-bit vectors, and vectors with identical binary codes in the sequence were collected. Conditional probability of the subsequent bit was ascertained, given the same preceding status (Figure 2). Sequences were allocated to different categories based on their conditional probabilities, meaning that sequences within a category shared identical conditional probabilities. Categories were established for all possible 4-bit sequences and were ranked according to their conditional probabilities. For example, the highest rank 3 signified the most complex pattern, whereas rank 1 indicated the most predictable pattern.

### Statistical analysis

Continuous variables with a normal distribution are expressed as mean ± standard deviation. Categorical variables are expressed as percentages. The demographics, comorbid diseases, and medication use of the surviving and expired patients were compared. Normality of the variables was tested using the Shapiro-Wilk test, and the between-group differences of continuous variables with normal distribution were tested using the Student’s t-test; the Mann-Whitney U test was used for non-normally distributed variables. Categorical variables were compared between the patients with different outcomes using Fisher’s exact test. The hazard ratios of variables with significant between-group differences were further estimated using the Cox proportional hazard regression model. The optimal dichotomization cutoff points of the variables were determined by the best results of the log-rank test sought from the 30th to 70th percentile in fifth percentile increments each time. All statistical analyses were performed using R software, version 3.5.0. Statistical significance was set at p < 0.05.

### Sample size calculation

The sample size for our study was determined through a power analysis. Previous research by Hynninen P. indicated a strong correlation between skin sympathetic nerve activity (SKNA) and sudomotor activity (Hynninen, 2020). Additionally, Syngle A et al. identified a large effect size in alterations of submotor activity after 12 weeks of DPP4i treatment (Syngle et al., 2021). In our study, we anticipated a medium to large effect size of 0.65 for changes in SKNA post-medication. We set the significance level (alpha) at 0.05 and aimed for a power of 85%, using a two-tailed paired t-test to evaluate this effect. Based on these parameters, the calculated sample size required for our study was determined to be approximately 44 participants per group.

## Results

We enrolled 96 patients in our study, general characteristics of whom are listed in Table 1. For treatment, 49 patients took SGLT2i, and 47 patients took DPP4i. No significant difference was seen in the underlying diseases, AC sugar, HbA1C, renal function, or medications (Table 2). Some patients had past medical history of heart failure but at the moment of study, they were asymptomatic, and their heart failure were heart failure with preserved ejection fraction or heart failure with recovered ejection fraction. No major adverse cardiovascular outcome occurred during this period, and no patient experienced urinary tract infections, pancreatitis, or hypoglycemic episodes. After 3 months of treatment, AC sugar and HbA1C decreased numerically (Table 2), though only in the SGLT2i group; the HbA1C reached statistical significance (for AC sugar, 138 ± 26 vs 126 ± 51, p = 0.154 for SGLTi group; 152 ± 55 vs 135 ± 48, p = 0.112 for DPP4i group; for HbA1C, 7.5 ± 1.3 vs 6.9 ± 1.0, p = 0.03for SGLT2i group, 7.8 ± 1.7 vs vs 7.1 ± 1.7, p = 0.07 for DPP4i group). Renal function was stable in both the groups during this period. During follow up, only one patient experienced major adverse cardiovascular outcome. The patient had ST-segment elevation myocardial infarction, which was treated by primary angioplasty. She was taking DPP4i for the treatment of diabetes mellitus, and has been doing well till date. HRV parameters between patients using SGLT2i and DDP4i exhibited no differences at baseline or after treatment, as shown in Supplementary Table S1. Additionally, there were no significant changes in HRV parameters from baseline to post-treatment in either group.

**TABLE 1:** Baseline characteristics of the study participants.

Basic characteristics	SLGT2i	DPP4i	P-value
	N = 49	N = 47	
Age (year)	68 ± 10	67 ± 14	0.82
Male, no. (%)	31 (63)	25 (53)	0.32
Atrial fibrillation, no. (%)	8 (16)	9 (19)	0.72
History of heart failure no. (%)	12 (24)	11 (23)	0.96
Hypertension no. (%)	43 (87)	42 (89)	0.80
Coronary artery disease no. (%)	24 (50)	27 (57)	0.41
Medication			
ACEi/ARB, %	35 (71)	27 (57)	0.76
Beta-blocker, %	33 (67)	33 (70)	0.86
Statins, %	30 (61)	25 (53)	0.43

Values are expressed as mean ± standard deviation, median (interquartile range), or number (percentage).

Abbreviations: SGLT2i, sodium-glucose co-transporter 2 inhibitor; DPP4i, Dipeptidyl peptidase-4, inhibitor; HbA1c, hemoglobin A1C; eGFR, estimated glomerular filtration rate; ACEi/ARB, angiotensin-converting enzyme inhibitor/angiotensin receptor blocker.

**TABLE 2:** Changes in clinical measurements.

Changes in parameters	Pre-treatment	Post-treatment	p
SGT2i group; N = 49			
AC sugar, mg/dL	138 ± 26	126 ± 51	0.154
HbA1C (%)	7.5 ± 1.3	6.9 ± 1.0	0.03
eGFR (ml/min/1.73 m^2^)	83 ± 27	83 ± 31	0.83
DPP4i group; N = 47			
AC sugar, mg/dL	152 ± 55	135 ± 48	0.112
HbA1C (%)	7.8 ± 1.7	7.1 ± 1.3	0.07
eGFR (ml/min/1.73 m^2^)	74 ± 30	77 ± 32	0.62

Abbreviations as Table 1.

**FIGURE 3:**
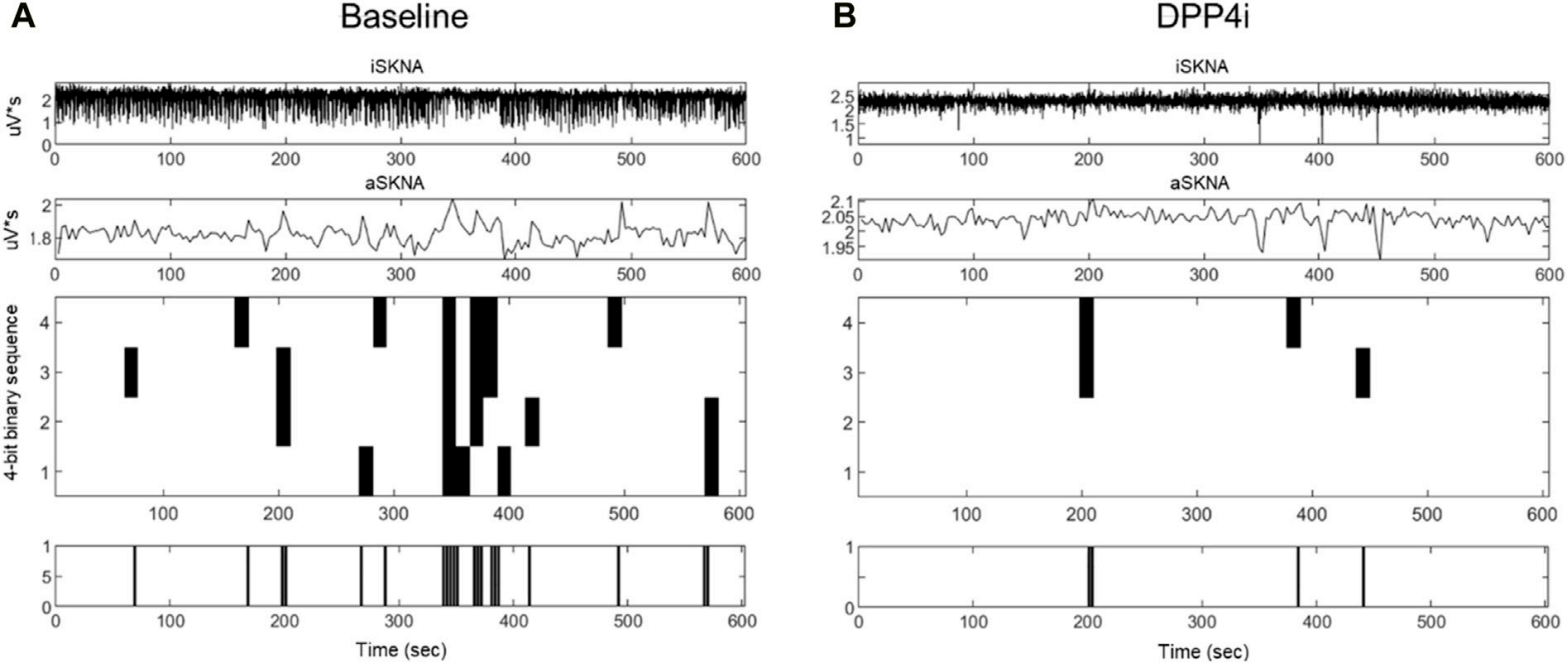
Typical characteristic activation patterns of aSKNA in patients before **(A)** and after **(B)** DDP4 inhibitor treatment. The traces from top to bottom represent iSKNA, aSKNA with a 3-s window, and 4-bit activation patterns, respectively. Each vertical line indicates a 4-bit binary sequence of activation patterns, with activation depicted in white and non-activation in black. Decreased activation frequency and an increase in less complex patterns (rank 1) were observed.

**FIGURE 4:**
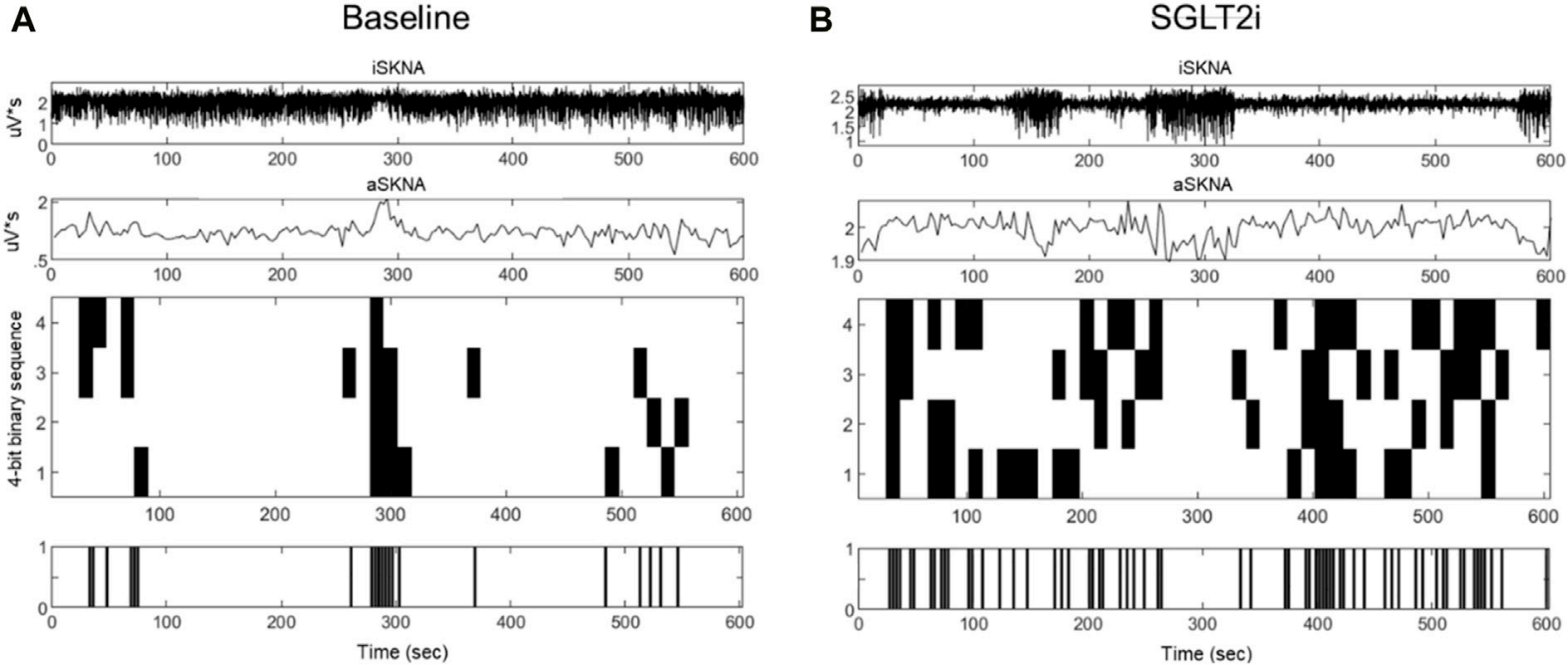
Typical characteristic activation patterns of aSKNA in patients before **(A)** and after **(B)** SGLT2 inhibitor treatment. The traces from top to bottom represent iSKNA, aSKNA with a 3-swindow, and 4-bit activation patterns, respectively. Each vertical line indicates a 4-bit binary sequence of activation patterns, with activation depicted in white and non-activation in black. Patients receiving SGLT2 inhibitors demonstrated patterns of greater complexity and extended durations of activation.

The results of aSKNA within a 3-s time window for each group, both before and after treatment, are presented in Table 3. Additionally, we compared the changes in the derived parameters before and after treatment in the SGLT2i and DPP4i groups to assess the direction and magnitude of the effects of the drugs on these parameters. Figures 3, 4 display the typical changes observed in the burst and symbolic entropy analyses of SKNA in patients treated with DDP4i and SGLT2i, respectively. Table 3 shows that at baseline, all aSKNA parameters measured over a 3-s window were similar between the groups. After a 3-month treatment period, the firing frequency significantly increased in SGLT2i inhibitor group, but decreased in the DDP4i group (baseline firirng frequency: 0.091 ± 0.048 vs 0.091 ± 0.038, p = 0.972; post treatment 0.104 ± 0.045, 0.083 ± 0.033; difference 0.013 ± 0.051 vs. −0.007 ± 0.049, p = 0.044 in SGLT2i and DPP4i group respectively). Furthermore, post-treatment measurements indicated a longer firing duration in the SGLT2i group (baseline 6.309 ± 4.266 vs 6.274 ± 3.125, p = 0.963; post treatment 7.34 ± 3.66 vs 5.906 ± 2.921, p = 0.034 in SGLT2i and DPP4i group respectively). However, no significant change was observed in other SKNA parameters following the treatments.

**TABLE 3:** aSKNA in 3-s time window.

SKNA	SGLT2i	DPP4i	P-value
	N = 49	N = 47	
Pre-treatment			
Baseline (μV)	1.812 ± 0.159	1.837 ± 0.121	0.388
Threshold (μV)	1.909 ± 0.123	1.928 ± 0.096	0.401
Frequency (b/m)	0.091 ± 0.048	0.091 ± 0.038	0.972
Duration (%)	17.819 ± 14.207	15.849 ± 6.658	0.388
Duration, long (%)	6.309 ± 4.266	6.274 ± 3.125	0.963
Duration, short (%)	11.511 ± 14.696	9.575 ± 6.493	0.408
Burst amplitude (μV)	1.863 ± 0.129	1.877 ± 0.112	0.564
Mean amplitude (μV)	1.948 ± 0.098	1.961 ± 0.083	0.477
Area (μV*mins)	0.328 ± 0.805	0.199 ± 0.158	0.286
Post-treatment			
Baseline (μV)	1.798 ± 0.198	1.839 ± 0.108	0.217
Threshold (μV)	1.894 ± 0.168	1.937 ± 0.083	0.116
Frequency (b/m)	0.104 ± 0.045	0.083 ± 0.033	0.011*
Duration (%)	17.947 ± 6.322	16.491 ± 8.343	0.325
Duration, long (%)	7.34 ± 3.66	5.906 ± 2.921	0.034*
Duration, short (%)	10.606 ± 6.476	10.585 ± 9.224	0.989
Burst amplitude (μV)	1.841 ± 0.192	1.886 ± 0.092	0.15
Mean amplitude (μV)	1.932 ± 0.146	1.969 ± 0.076	0.121
Area (μV*mins)	0.228 ± 0.138	0.245 ± 0.264	0.681
Difference			
Baseline (μV)	−0.013 ± 0.221	0.002 ± 0.106	0.678
Threshold (μV)	−0.015 ± 0.185	0.009 ± 0.089	0.584
Frequency (b/m)	0.013 ± 0.051	−0.007 ± 0.049	0.044*
Duration (%)	0.128 ± 15.229	0.642 ± 10.086	0.954
Duration, long (%)	1.032 ± 4.383	−0.368 ± 3.834	0.113
Duration, short (%)	−0.904 ± 15.873	1.009 ± 9.797	0.698
Burst amplitude (μV)	−0.022 ± 0.196	0.008 ± 0.098	0.438
Mean amplitude (μV)	−0.016 ± 0.155	0.008 ± 0.085	0.475
Area (μV*min)	−0.1 ± 0.821	0.046 ± 0.232	0.409

All values are expressed as mean ± SD. *denotes p < 0.05.

Abbreviations: SKNA, skin sympathetic nerve activity; please refer to text for definition.

By symbolic entropy analysis (Table 4), no significant difference was found between the DDP4i and SGLT2i groups before and after treatment in terms of the components of the most predictive to complex patterns (Ranks 1–3). However, the most complex patterns (Rank 3) were significantly higher post-SGLT2i treatment than post-DDP4i therapy (0.084 ± 0.028 vs 0.07 ± 0.024, p = 0.01). Furthermore, the direction of change in Rank 3 following SGLT2i treatment was opposite to that observed in the DDP4i group (0.012 ± 0.036 vs. −0.005 ± 0.037, p = 0.024). Representative figures describing pre-and post treatment changes in DPP4i and SGLT2i were showed in Figures 3, 4 respectively.

**TABLE 4:** Entropy parameters.

SKNA	SGLT2i	DPP4i	P-value
	N = 49	N = 47	
Pre-treatment			
Rank 1	0.737 ± 0.123	0.736 ± 0.104	0.981
Rank 2	0.192 ± 0.096	0.189 ± 0.081	0.853
Rank 3	0.071 ± 0.031	0.075 ± 0.026	0.497
Post-treatment			
Rank 1	0.702 ± 0.117	0.756 ± 0.088	0.011*
Rank 2	0.215 ± 0.092	0.174 ± 0.067	0.015*
Rank 3	0.084 ± 0.028	0.07 ± 0.024	0.01*
Difference			
Rank 1	−0.035 ± 0.138	0.02 ± 0.138	0.088
Rank 2	0.023 ± 0.106	−0.014 ± 0.104	0.146
Rank 3	0.012 ± 0.036	−0.005 ± 0.037	0.024*

All values are expressed as mean ± SD. *denotes p < 0.05.

Abbreviation: HR, heart rate.

## Discussion

In this study, we reported that neuECG is a feasible tool for evaluating sympathetic nerve activity in patients with DM in an outpatient clinical setting. After a 3-month treatment with SGLT2 inhibitors, there was a notable decrease in firing frequency and a significant increase in the duration of long firing episodes using 3-s aSKNA window. Entropy analysis revealed that complex activation patterns may indicate effective modulation dynamics in the SGLT2 inhibitor group. The above findings supported the sympathetic modulation of these drugs, provided a pharmacological basis for favorable cardiovascular and renal outcomes in patients taking SGLT2i, and showed the powerful application of neuECG in delineating changes in the autonomic nervous system.

Autonomic nervous system (ANS) activation plays a crucial role in cardiovascular outcomes (Karakas and Koenig, 2013), including myocardial infarction, heart failure (Wu and Vaseghi, 2020), and arrhythmia initiation and termination (Kusayama et al., 2019; Tsai et al., 2023). Compared to age-matched healthy controls (Supplementary Table S2), DM patients displayed significantly higher aSKNA threshold, mean amplitude, and burst amplitude over a timescale of 1–5 s. This suggests marked autonomic dysfunction in individuals with DM. In our previous study, we found that in patients undergoing ablation for atrial fibrillation, post-ablation firing for a long duration in the aSKNA 5-s window was a significant predictor of recurrence (Chen et al., 2022). Patients free of AF after ablation had a longer firing duration in the 5-s aSKNA analyses. Although the mechanism of recurrence was complex, given that higher SNS activity initiated AF, we believe that proper modulation of the autonomic nervous system is crucial for AF treatment. Likewise, we found that treatment with SGLT2i had similar effects on the SNS, which might explain the effects of SNS modulation translating into favorable cardiovascular outcomes.

The onset of T2DM is becoming earlier in population (Pan and Jia, 2018). In addition to the consequences of hyperglycemia, T2DM has severe adverse cardiovascular and renal outcomes (Lo et al., 2020), and SGLT2i could be a potential medication to reverse these outcomes (Zelniker et al., 2019). Our study provided solid evidence for SNS modulation by SGLT2i in patients with DM. In our previous studies, SNS played a crucial role in determining mortality in critically ill patients (Chen et al., 2021) and could predict AF ablation outcomes (Chen et al., 2022). We believe that we provided mechanistic evidence to treat young patients with SGLT2i, which might improve their health condition, especially CV and renal function. Syngle A et al. observed significantly higher sudomotor activity after 12 weeks of DPP4 inhibitor treatment, indicating an increase in sympathetic activity. Additionally, there were significant reductions in both blood pressure and heart rate responses to standing and the Valsalva maneuver compared to baseline measurements ((Syngle et al., 2021). In our study, we found that HRV parameters were less sensitive in a resting state compared to their response under stimuli. Moreover, most HRV parameters did not show significant changes in patients treated with SGLT2i, even after 1 year (Sardu et al., 2022). Given the strong association between SKNA and sudomotor activity, SKNA may serve as a more sensitive surrogate for evaluating autonomic function.

Entropy analysis was used to assess the predictability and dynamics of neural activities. Research has demonstrated that a reduction in entropy may serve as an indicator of various diseases, including cardiovascular diseases (Costa et al., 2005; Ho et al., 2011; Chiu et al., 2017) and type I diabetes (Trunkvalterova et al., 2008). The observed increase in the most complex patterns (Rank 3) following SGLT2 inhibitor (SGLT2i) treatment, as compared to DDP4 inhibitor (DDP4i) therapy, not only indicated an increase in the complexity of autonomic nerve activity but also pointed to an adaptive improvement in modulation dynamics following SGLT2i treatment. This was in addition to the changes in firing characteristics, such as frequency and duration. These findings go beyond cardiac autonomic regulation, suggesting a wider physiological and therapeutic importance of SGLT2 inhibitors. These insights underscored the importance of incorporating symbolic entropy analysis into the assessment of neuECG dynamics to provide a deeper understanding of the behavior of the autonomic nervous system in health and disease. Our findings indicate that the complexity of autonomic system modulation can be quantified using entropy analysis. Furthermore, the cardio-renal protective effects of SGLT2 inhibitors may be attributed to the restoration of autonomic modulation in patients with DM.

SGLT2i show some contraindications in patients with ketoacidosis, advanced chronic kidney disease (CKD), or frequent urinary tract infection after treatment with SGLT2i (Seidu et al., 2022). However, in continuous usage of SGLT2i in advanced CKD DPP4i has been advocated (Yau et al., 2022). DPP4i, especially linagliptin, can be used in all patients, including those with liver or renal dysfunction and DM, except for patients with pancreatitis after DPP4i (Scott, 2011). From pharmacological point of view (including SNS modulation and other effects) and current clinical evidence, given that SGLT2i reduced major cardiovascular events and improved renal outcome in patients with T2DM or heart failure regardless of glycemic control, and based on our data from neuECG analyses on autonomic modulation, we would recommend SGLT2i as a second-line medication or even first-line medication in patients with T2DM and as a standard treatment in patients with HF.

This study has several limitations. The number of recruited patients was limited, and the recording was performed at an outpatient clinic. Environmental and seasonal effects cannot be ignored. While the study period was 3 months only, the patients used the drugs continuously and could have changed to combination therapy (SGLT2i and DPP4i). However, the long-term effects of these drugs and their combinations would require further elucidation. All patients were managed by a single cardiologist and, although the number of cases was limited, the treatment policies for their underlying diseases were similar. We believe that unnecessary changes in medication or other interventions that interfere with the outcomes in different scenarios can be avoided. We intentionally performed a randomized study; however, the use of SGLT2i and DPP4i had compelling indications or contraindications and there are complex reimbursement issues in Taiwan (approval of SGLT2i for heart failure were later than publish of randomized controlled trials and international guidelines). Moreover, during the study period, new clinical evidences for SGLT2i were published. Therefore, this study was not fully randomized. We belived that HbA1C/Age/Sex/Ethnicity might affect autonomic function. The study population were all East-Asian and their age/sex and baslineline HbA1C were matched in study groups. We found there’s no difference in SKNA parameters between men and women except firing frequency (Supplementary Table S3). However futher large scale study might be conducted to address these concerns. Our study showed entropy analysis was a powerful tool to evaluate autonomic function in DM patients; however, we were not sure the application could be generalized to other diseases.

In conclusion, our study demonstrated that improved autonomic regulation, as evidenced by SKNA analyses, including burst and symbolic entropy analyses, could be a plausible explanation for the favorable cardiovascular and renal outcomes associated with SGLT2i observed in numerous clinical trials. Furthermore, we refined neuECG analyses to enhance the ability of the method to evaluate autonomic modulation under different scenarios. We would recommend considering SGLT2i as a second- or even first-line medication for patients with type 2 diabetes mellitus (T2DM), even in the absence of heart failure, unless there are compelling contraindications.

## Data Availability

The raw data supporting the conclusions of this article will be made available by the authors, without undue reservation.

## Abbreviations

T2DM, type 2 diabetes mellitus; SGLT2i, sodium glucose co-transporter 2 inhibitor; DPP4i, Dipeptidyl peptidase-4 inhibitor; CV, cardiovascular; SNS, sympathetic nervous system; AMI, acute myocardial infarction; HRV, heart rate variability; ECG, electrocardiography; SKNA, sympathetic nerve activity; iSKNA, integrated SKNA; aSKNA, averaged iSKNA; HbA1C, hemoglobin A1C; Hz, Hertz; ANS, autonomic nervous system; AF, atrial fibrillation.
