# Supplementary material for: Autonomic modulation by SGLT2i or DPP4i in patients with diabetes favors cardiovascular outcomes as revealed by skin sympathetic nerve activity
Source: Front Pharmacol. 2024 Jul 30;15:1424544. doi: 10.3389/fphar.2024.1424544 (PMC11319125; doi:10.3389/fphar.2024.1424544)
Supplement: Supplementary file 1 [file Table1.pdf]

Supplement Table 1 Heart rate variability parameters

HRV	SGLT2i N = 49	DPP4i N = 47	*P* value
Pre-treatment			
Mean	815.702±95.785	829.302±121.732	0.57
HR	74.556±8.732	73.958±11.257	0.78
SDRR	21.978±11.894	21.907±10.548	0.97
RMSSD	18.115±16.094	16.87±10.8	0.68
pNN50	4.497±10.744	2.692±6.974	0.36
pNN20	18.423±21.205	22.334±21.243	0.39
P_VLF	247.992±237.579	303.524±283.044	0.32
P_LF	142.435±225.624	111.391±100.368	0.42
P_HF	114.704±244.964	100.618±130.055	0.74
Post-treatment			
Mean	850.849±101.948	819.01±122.947	0.19
HR	71.569±8.944	74.936±11.527	0.12
SDRR	24.124±14.597	24.194±14.792	0.98
RMSSD	18.908±13.535	18.417±13.673	0.86
pNN50	3.923±8.41	3.492±7.495	0.80
pNN20	20.086±18.486	21.863±20.982	0.67
P_VLF	384.231±578.563	377.644±497.348	0.95
P_LF	216.905±390.315	190.922±421.797	0.76
P_HF	121.305±227.311	132.173±254.651	0.83
Difference			
Mean	31.466±104.269	-7.373±107.004	0.06
HR	-2.7±9.486	0.704±9.937	0.08
SDRR	2.133±12.861	1.133±13.991	0.30
RMSSD	0.265±11.857	0.229±12.692	0.88
pNN50	-0.802±8.538	0.101±9.139	0.55
pNN20	1.503±18.447	-1.517±22.058	0.60
P_VLF	145.563±593.913	63.868±554.304	0.12
P_LF	78.447±322.287	36.493±306.013	0.13

P_HF	4.53±157.244	-1.456±153.609	0.85

Please refer to reference for definition of parameters

Refence

1. Heart rate variability: standards of measurement, physiological interpretation and clinical use. Task Force of the European Society of Cardiology and the North American Society of Pacing and Electrophysiology. Circulation 1996;93(5):1043-65.

Supplement table 2: SKNA parameters in DM patients vs. healthy control

Parameters	DM (n=96)	Control (n=26)	*p* value
Tranditional parameters			
Baseline (μV)	1.825±0.14	1.779±0.098	0.058
Threshold (μV)	1.919±0.109	1.875±0.082	0.027
Frequency (b/m)	0.091±0.043	0.092±0.035	0.882
Duration (%)	16.775±10.864	15.942±4.993	0.571
Duration, long (%)	6.29±3.686	6.365±2.975	0.913
Duration, short (%)	10.485±11.11	9.577±4.066	0.508
Mean amplitude (μV)	1.871±0.12	1.818±0.095	0.022
Burst amplitude (μV)	1.955±0.09	1.91±0.074	0.011
Area (μV*mins)	0.26±0.564	0.2±0.073	0.306
Entropy parameters			
Rank 1	0.736±0.113	0.739±0.091	0.918
Rank 2	0.19±0.088	0.19±0.072	0.990
Rank 3	0.073±0.028	0.071±0.021	0.639

Please refer to text for definition of parameters

Supplement table 3: SKNA parameters between sex

Parameters	Male (n=76)	Female (n=20)	*p* value
Tranditional parameters			
Baseline (μV)	1.824 + 0.139	1.81 + 0.155	0.737
Threshold (μV)	1.915 + 0.114	1.911 + 0.114	0.842
Frequency (b/m)	0.102 + 0.045	0.078 + 0.039	0.031
Duration (%)	16.577 + 6.038	17.361 + 16.284	0.116
Duration, long (%)	7.087 + 3.93	5.514 + 3.296	0.060
Duration, short (%)	9.49 + 5.244	11.847 + 16.925	0.296
Mean amplitude (μV)	1.864 + 0.13	1.867 + 0.115	0.869
Burst amplitude (μV)	1.949 + 0.102	1.954 + 0.081	0.789
Area (μV*mins)	0.203 + 0.132	0.369 + 0.923	0.299
Entropy parameters			
Rank 1	0.712 + 0.116	0.763 + 0.108	0.103
Rank 2	0.21 + 0.093	0.168 + 0.081	0.088
Rank 3	0.078 + 0.027	0.069 + 0.03	0.399
